# Evaluation of Environmental Sampling for Detection of *Mycobacterium avium* subspecies *paratuberculosis* in the Pre-Weaned Calf Area and Calving Area of Infected Dairy Farms Enrolled in a Voluntary Johne’s Disease Control Programme

**DOI:** 10.3390/ani13040669

**Published:** 2023-02-14

**Authors:** Niamh L. Field, John F. Mee, Conor G. McAloon

**Affiliations:** 1 Animal and Bioscience Research Department, Teagasc, Moorepark Research Centre, P61 P302 Fermoy, Ireland; 2 UCD School of Veterinary Medicine, University College Dublin, D04 W6F6 Dublin, Ireland

**Keywords:** paratuberculosis, environmental, PCR detection, transmission, risk

## Abstract

**Simple Summary:**

Transmission of Mycobacterium avium subspecies paratuberculosis (MAP) from infected adult cattle to susceptible calves occurs mainly through contamination of the calf environment, i.e., the calving pen and the pre-weaned calf rearing area, with adult faeces. Control programmes for Johne’s disease utilise a risk assessment and management plan (RAMP) to evaluate the risk of transmission of infection on a specific farm, however the assessment is limited by its subjective, point-in-time nature. The objective of this study was to evaluate a novel environmental sampling protocol for measurement of MAP contamination of the calf environment on infected farms. Across 28 infected farms, 46% had detectable MAP in the calf environment, with 21% of farms having detectable MAP in the pre-weaned calf area. There was no significant association found between the RAMP scores for each farm and the result of environmental testing for MAP, and there was a moderate correlation found between RAMP scores and the level of MAP contamination as quantified by PCR. We conclude that environmental sampling is a potentially useful tool to objectively measure transmission risk in the calf environment on farms, as a complement to the annual RAMP.

**Abstract:**

One of the pillars of Johne’s disease control is to break the cycle of transmission from infectious adult animals to young susceptible animals. Many control programmes utilise a risk assessment and management plan (RAMP) to identify specific risks for transmission of infection on individual farms and then recommend tailored biosecurity measures to mitigate the risk. It is important that infected farms in particular, practice effective biocontainment in the calving area and pre-weaned calf area. The objectives of this study were: (1) to determine what proportion of MAP-infected farms (PCR-positive) enrolled in a control programme had detectable MAP in their calf environment; (2) to compare RAMP scores and apparent within-herd prevalence (aWHP) of PCR-positive and PCR-negative farms; (3) to evaluate the correlation between RAMP scores, aWHP and levels of contamination based on PCR test Ct value. A novel environmental sampling protocol combining manure samples and boot swab samples was conducted in the calving area and pre-weaned calf area on 28 dairy farms with PCR-confirmed MAP infection. All samples were tested with PCR. Logistic regression modelling was used to evaluate the association between RAMP score and aWHP and PCR outcome. Overall, 46% of farms had positive PCR samples in either the calving area, pre-weaned calf area or both. The calving area was positive in 36% of farms and the pre-weaned calf area was positive in 21% of farms. There was a moderate, negative correlation (r = −0.45; *p* = 0.08) observed between RAMP scores and Ct value at the sample-level that slightly missed the required significance level. No significant association was identified between RAMP scores or aWHP and PCR test outcome (positive or negative). It was concluded that environmental sampling of the calving area and pre-weaned calf area has potential applications in the assessment of transmission risk on infected farms and could be used to monitor the efficacy of biosecurity measures over time. Further research conducted on a larger scale is required to support these results.

## 1. Introduction

Johne’s disease is a bacterial infection of cattle and other ruminants caused by *Mycobacterium avium* subspecies *paratuberculosis* (MAP). MAP is an obligate intracellular pathogen incapable of environmental proliferation; however, the organism is sufficiently robust to survive in the environment for prolonged periods [1]. MAP is primarily transmitted orally through ingestion of faeces, colostrum or milk from an infectious animal [2]. Environmental contamination of housing and pasture with faeces from infected animals constitutes a risk for transmission of infection to susceptible animals. Cattle are usually infected as calves and develop clinical signs as adults, such as reduced productivity, progressive diarrhoea, and weight loss [2]. MAP is of particular significance in dairy herds due to impacts on animal health, associated production losses [3,4,5], and controversial associations with Crohn’s disease in humans [6]. Even without evidence of a causal link, the association between MAP and Crohn’s disease is likely sufficient to affect consumer confidence in dairy products.

Control programmes for Johne’s disease usually involve a combination of herd testing to detect infection and biosecurity measures to reduce the spread of disease within herds and between herds [7,8]. Test and cull strategies have been shown to be ineffective when used in isolation to control the disease in infected herds [9,10], due to the poor sensitivity of diagnostic tests in individual animals. Therefore, the emphasis must be on breaking the cycle of transmission of MAP from potentially infectious animals to susceptible animals. Faeces is the most important source of transmission [11], both through faecal contamination of the calf environment and through faecal contamination of colostrum and milk fed to calves [12]. Direct shedding of MAP in milk and colostrum is of lesser significance, occurring mainly in clinically affected animals, and at low-levels in a small proportion of subclinical animals [13,14].

Many control programmes internationally use variations of a risk assessment and management plan (RAMP) in herds to reduce the risk of transmission of MAP on infected farms [7,8,15]. The risk assessment (RA) portion of the RAMP aims to score the risk of transmission of MAP within the herd, while the management plan formulated on completion of the RA recommends risk mitigation measures to address farm-specific risks identified during the RA that may lead to the exposure of susceptible calves to potentially infectious faeces, milk and colostrum. A combination of test-and-cull and biocontainment measures have been shown to be most effective to reduce MAP infections [9]. These measures include removing the calf from the dam immediately after birth, strict hygiene of calving pens, feeding of milk replacer instead of whole milk and preventing contamination of calf rearing areas with adult manure. Limitations to the RAMP include a reliance on farmer recollections and reporting of biocontainment actions, as well as the “point-in-time” nature of the observations of the veterinarian conducting the RA. Additionally, compliance with the management plan has been found to be poor [16,17] and implementation is inadequately assessed with indirect monitoring structures. Testing of adult animals and comparison of consecutive risk assessments remain the only way of measuring and comparing progress over time [18,19].

Environmental sampling may be a potentially useful technique to detect MAP in the pre-weaned calf area and calving area of farms. Boot swabs have been evaluated as a practical and efficient way of obtaining environmental samples for MAP testing in adult cow housing on farms and have shown sensitivity that is comparable to taking standard grab samples of manure [20,21]. Boot swabs have also been used successfully in poultry flocks to test bedding material in housing for the presence of *Salmonella* [22,23], with boot swab kits commercially available for screening flocks. Calving pens often have areas of manure concentration near the feeding area, suitable for grab samples, as well as bedded areas more suited to boot swabs. Boot swabs are likely most suitable for sampling calf pens which typically have dry bedding such as straw or peat. A blended testing strategy combining standard environmental manure sampling and boot swab sampling may be effective and practical for objectively monitoring the efficacy of biocontainment measures recommended during a RAMP. Our primary hypothesis was that detection of MAP in the calf environment is associated with transmission risk (as measured during the RAMP) and apparent within-herd prevalence (aWHP) of infection. Our secondary hypothesis was that the level of contamination in a sample (as measured by the Ct value of the PCR test) is correlated with aWHP and RAMP score. The objectives of this study were (1) to determine what proportion of MAP-infected farms enrolled in a control programme had detectable MAP in their calf/calving environment, (2) to compare RAMP scores and apparent within-herd prevalence (aWHP) of PCR-positive and PCR-negative farms, (3) to evaluate the correlation between RAMP scores, aWHP and levels of contamination based on PCR test Ct value.

## 2. Materials and Methods

### 2.1. Herd Selection

In a previous study (unpublished) conducted in 2019, 122 dairy herds were selected from the Irish Johne’s Control Programme (IJCP) database using a stratified random selection process. Briefly, the stratification criteria used for the previous study was based on the MAP PCR status of the herds to allow a minimum proportion of confirmed-infected herds to be selected. The IJCP is a voluntary control programme open to both dairy and beef herds that aims to identify MAP-infected herds and control the spread of Johne’s disease both between herds and within herds. Herds registered in the programme conduct annual herd testing for MAP using individual serum or milk ELISA in addition to annual RAMPs conducted by private veterinary practitioners. In total, 1658 dairy herds were registered in the IJCP at that time. From this sample of 122 herds, 39 herds with confirmed infection based on individual faecal PCR were identified. The herds all had MAP infection confirmed within the last five years. A total of 35 herds were randomly selected from this cohort using the Excel random number generator, as the maximum number of herds it would be possible to visit for sampling during the spring calving period. Of these, 28 farmers agreed to participate in sample collection during the spring period of 2022. These were all spring calving herds, located mostly in the south and east of Ireland. Herd size ranged from 41-393 cows with a mean herd size of 166 at the time of sampling.

### 2.2. RAMP Scores

Herds registered in the IJCP undergo a RAMP annually [18]. It is completed by an approved veterinary practitioner (AVP) who has undergone specific training in Johne’s disease control and management. The RAMP comprises a non-scored bioexclusion section, and four scored sections: pre-weaned heifers risk assessment (scored out of 80), heifers over six months old risk assessment (scored out of 33), cows risk assessment (scored out of 34) and calving area risk assessment (scored out of 80) (template available on request). The higher the score assigned for an area, the greater the perceived risk of transmission of MAP.

The AVP uploads the scores to an online database hosted by the Irish Cattle Breeding Federation (ICBF), from which, with authorisation from the farmer, the first author downloaded information for each of the study herds.

### 2.3. Apparent Within-Herd Prevalence

The apparent within-herd prevalence (aWHP) for each herd was calculated based on the most recent annual whole-herd ELISA test recorded on the IJCP database. Herds registered in the IJCP must have all animals over two years old tested with MAP ELISA using either serum or milk samples. aWHP was calculated as the percentage of total animals tested by ELISA that had a positive or inconclusive result, as this is the procedure followed in the IJCP.

### 2.4. Sampling

All 28 herds were visited once between February and March 2022 during the spring calving period. The boot swabs used were sourced from Trafalgar Scientific Ltd., 190 Waterside road, Hamilton Industrial Estate, Leicester, LE5 1QZ, and were produced specifically for *Salmonella* sampling of poultry housing. Each boot swab pair consisted of two plastic overboots and two absorbent boot swabs pre moistened with maximum recovery diluent to aid recovery of organisms. On each farm three boot swab samples were obtained, one pair from the calving area and two pairs from the pre-weaned calf area. The procedure used for boot swab sampling was adapted from Eisenberg et al. (2013). Fresh overboots were used between the two locations. There was some variation between farms in the type of calving pens (individual, group, combination) and calf pens (size, floor type, number of pens) but at each location the same sampling protocol was used. Fresh overboots and boot swabs were put on at the entrance to the pen/shed, any alleyway/walkway in front of the pen was walked first, followed by entry into the pen, and then the pen was thoroughly walked in a zigzag pattern to maximise the surface area sampled. In the case of more than two separate calf sheds, the total area was roughly divided in two parts (two boot swab pairs) and boot swabs and overboots removed to walk across to the next shed before putting them back on to continue sampling. The same protocol was used for more than one calving pen. After sampling, the boot swabs were double-bagged in plastic zip-lock bags with samples from the same location pooled together to form one sample. All boot swab samples were placed in a −20 °C freezer within 48 h.

A manure sample was also obtained where possible off the floor of the calving pen, using the procedure for sample collection described by Wolf et al. (2014) [24]. Briefly, the pooled sample consisted of at least four “grabs” of mixed manure from separate sites in the pen, typically at the feeding area or around water troughs. The manure was mixed thoroughly in a mixing bag before transferring 20 g into a sample pot, which was kept refrigerated (4 °C) until it was tested in the laboratory.

### 2.5. Boot Swab Sample Processing

The boot swabs were defrosted just prior to processing in the laboratory. The protocol for processing was based on the method described by Eisenberg et al. (2013) with modifications made to accommodate test kit requirements, including standardising dilution rates of boot swab samples with varying weights of faecal material. Once defrosted, each boot swab was placed in a paddle blender bag and allowed to settle for 20 min at room temperature. The weight of boot swabs was subtracted from the total weight to give a faecal weight for each sample. The maximum volume of diluent that could be added to any sample was 150 mL (for sample handling reasons) and samples had to be diluted at a ratio of 6.67 mL to 1 g of faeces, (as required by the kit and in order to ensure results were comparable); therefore, the maximum faecal weight that could be processed as a single sample was 22.5 g.

Samples with a faecal weight of greater than 22.5 g had to be divided and processed as separate sub-samples. Demineralised water was added to each sub-sample in a weight of faeces to volume of water ratio of approximately 1: 6.67. Demineralised water was massaged into the boot swab sub-sample by agitating the bag, then allowing it to rest for 5 min. Each sub-sample was paddle-blended for 3 min. The recovered liquid was retrieved from the boot swab sub-sample and transferred to a sterile collection container. Particular attention was given to squeezing out the material to allow maximum recovery. The liquid sample was allowed to rest for 15 min to facilitate sample separation. Ten ml of supernatant was loaded into the ADIAfilter (Bio-X Diagnotics). The sample was centrifuged for 5 min at 3000× *g* and supernatant discarded. A volume of 500 µL of demineralised water was added to the recovered sample pellet, vortexed for 15 s then transferred to a lysis beads tube. The sample was disrupted in a bead-beater three times for 45 s each time with a 5-min rest period between each beading phase. After mechanical disruption the sample was centrifuged for 5-min at 15,000× *g* and 220 µL of the resultant supernatant phase was removed and was ready for the MAP DNA extraction protocol.

### 2.6. DNA Extraction and Amplification

The supernatant from the boot swab sub-samples and the manure sample from the calving pen floor underwent a multi-step DNA extraction process suitable for MAP. Briefly, samples were suspended in buffer then cleaned and concentrated via the use of the ADIAfilter (Bio-X Diagnotics) and centrifugation for 5 min at 3000× *g* to create a bacterial pellet. Physical disruption of the bacterial pellet was achieved via beating with glass beads which results in the release of DNA. A commercial DNA extraction kit QIAmp^®^ DNA Mini Kit (Qiagen) was then used to extract, clean and concentrate the DNA for use in the PCR reaction. The ADIAVET™ Paratb RealtimePCR kit (Bio-X Diagnotics) which detects the target gene IS900 was used for the detection of MAP DNA. In order to ensure the validity of results, four controls were included in each PCR run. A negative control was used to demonstrate that contamination of reagents or test wells did not occur during the process. An external positive control was added to each sample prior to DNA extraction to confirm that the DNA extraction process worked. A single kit positive control was included in each run to confirm that DNA amplification occurred. A validation control, composed of extracted DNA from a previously positive faecal sample, was included in every test run to challenge the cut-off of the method. The sample was considered negative if no Ct value was expressed or if greater than 45 Ct. The sample was considered inconclusive if Ct >36 as weak positive samples were not always reproducible after this value. The sample was considered positive if Ct ≤ 36.

### 2.7. Data Analysis

The results of testing are reported at sub-sample-level, sample-level (pre-weaned calf area, calving area) and farm-level, initially including negative, inconclusive and positive results but subsequently results were dichotomised so that inconclusive results were treated as negative. In order to obtain sample-level PCR results i.e., one pre-weaned calf area result and one calving area result per farm, sub-sample results for each location were pooled, including the floor samples from the calving area, such that a location was deemed to be PCR-positive if at least one sub-sample from the location was positive on PCR (Ct ≤ 36). For farm-level analysis, a farm was defined as PCR-positive if there was at least one positive PCR result for either calf pens or calving pens.

Logistic regression models were built with four different outcomes, categorized as follows: (1) farm-level PCR outcome; (2) pre-weaned calf area PCR outcome; (3) calving area PCR outcome; and (4) sample-level PCR outcome (all PCR results from both locations included). RAMP scores (overall score, pre-weaned calf area score or calving area score, as appropriate) and aWHP were the independent variables of interest. Model 4 had two observations per farm, so farm was included as a random effect. Variables were visually assessed for normality of distribution using histograms prior to inclusion in the models. Inconclusive results were defined as negative for the purpose of modelling.

Spearman’s correlation test was used to evaluate associations at sample-level between location-specific RAMP scores and the lowest Ct value at the location, and aWHP and the lowest Ct values, in PCR-positive farms. High correlations were deemed as coefficients between 0.5–1, medium correlations as coefficients between 0.3–0.49, low correlations as coefficients below 0.30. With varying numbers of samples tested between farms for each location, the most biologically accurate method of comparing contamination quantitatively was to select the lowest Ct value recorded for each location on each farm.

## 3. Results

### 3.1. Apparent Within-Herd Prevalence

In total, 26 herds had serology results available to allow calculation of aWHP (Table 1). There were 14 farmers that used predominantly blood samples and 12 used predominantly milk samples for their herd test. The most recent blood/milk test results for study herds ranged between one and six months before or after the time of boot swab sampling. The aWHP for the herds ranged from 0–21%, with a median of 3.5%. The mean and median aWHP for PCR-positive and PCR-negative farms was 5.1% and 5%, and 4.8% and 3%, respectively, with no significant difference identified between groups (*p* = 0.48).

### 3.2. Environmental Samples

Calving area floor samples were obtained for 23 out of 28 herds. Eight floor samples were positive, four were inconclusive and eleven were negative. Five farms had no manure available in the pens to sample. In total, 84 boot swab pairs were obtained from all 28 farms (three pairs per farm). In total, 104 diluted faecal sub-samples for PCR extraction and amplification were obtained during processing of the boot swabs (2–7 per farm). Overall, 57 of these sub-samples were negative, 26 inconclusive and 21 positive. Table 2 shows the distribution of positive, negative and inconclusive results when boot swab sub-samples and calving area floor samples were pooled to give one result per location on each farm. 10/28 (36%) and 6/28 (21%) of farms had positive PCR results in the calving area and pre-weaned calf area, respectively. A total of 13/28 (46%) of farms had at least one positive sample from either location. There were 4/28 (14%) of farms that had at least one inconclusive sample from either location, and no positive samples.

### 3.3. RAMP Scores

**Figure 1:**
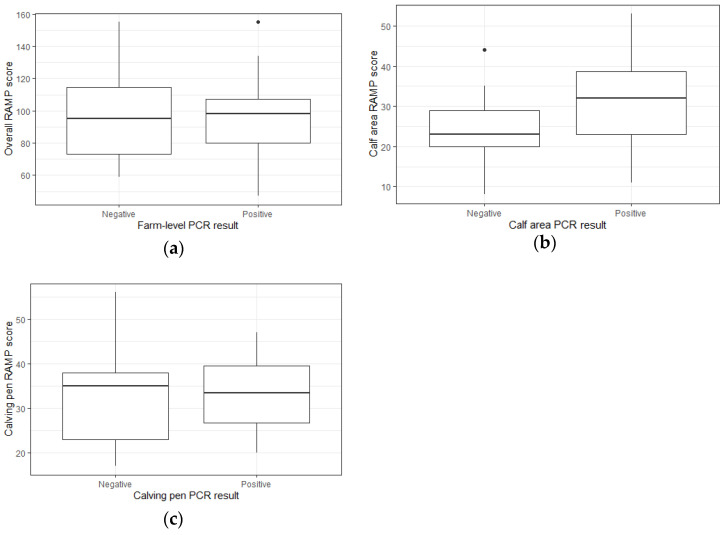
Boxplots illustrating the distribution of RAMP scores for the overall farm (**a**), pre-weaned calf area (**b**) and the calving area (**c**). The black dots in the graphs represent outliers in the data.

The most recent RAMP scores were available for 27 out of 28 herds and are described in Table 1. Overall RAMP scores ranged from 47–155 (out of total score of 227). The lower the score, the lower the perceived risk of transmission of MAP. The scores for the pre-weaned calf area ranged from 8 to 53, and for the calving area from 17–56 (each out of total 80). The mean overall RAMP score in PCR-positive and PCR-negative farms was 96.8 and 95.9, respectively, with no significant difference between mean scores (*p* = 0.93). The distribution of scores between both groups is illustrated in Figure 1.

The mean RAMP scores for calf pens across farms that were PCR-positive for calf pens and PCR-negative for calf pens were 31.5 and 23.7, respectively, with no significant difference between mean scores (*p* = 0.12). The distribution of scores between both groups is illustrated in Figure 1.

The mean RAMP scores for calving pens across farms that were PCR-positive and PCR-negative for calving pens was 33.2 and 33.9, respectively, with no significant difference between means (*p* = 0.85). The distribution of scores between both groups is illustrated in Figure 1.

### 3.4. Logistic Regression Models

A log transformation was applied to the aWHP data prior to modelling due to positive skewness. As one farm had an aWHP of 0, it was necessary to add a constant value (1) to aWHP prior to transformation, hence the variable included in the models was log(aWHP+1). RAMP score and aWHP were not significantly associated with PCR outcome in any of the models. The model results are summarised in Table 3.

### 3.5. Correlation Tests

**Figure 2:**
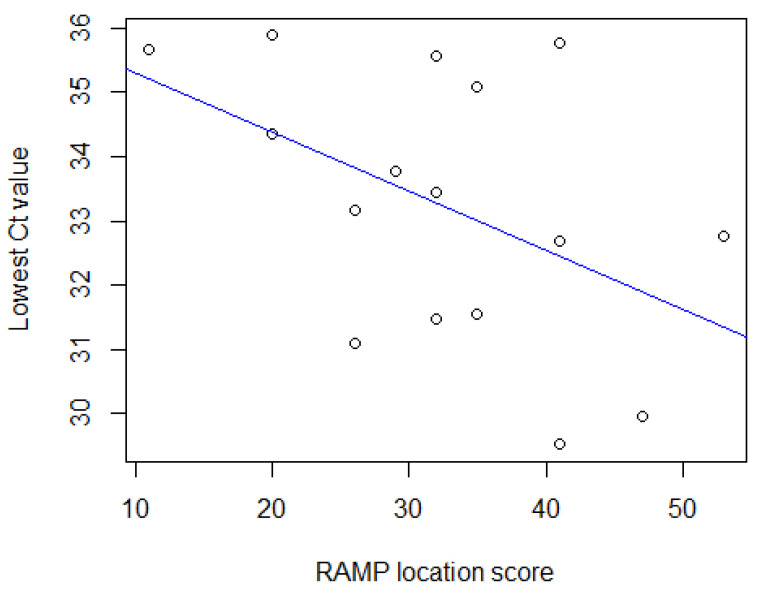
Scatterplot to illustrate the correlation between the lowest Ct value for a location and the RAMP score for the location.

The association between the lowest Ct value for a location and the RAMP score for that location was assessed by selecting all PCR-positive sample-level results (n = 16) from all farms. There was moderate negative correlation found using Spearman’s rank correlation coefficient (−0.45; (*p*-value = 0.08)) illustrated in Figure 2.

The association between the lowest Ct value for a location and aWHP was assessed by selecting all PCR-positive sample-level results (*n* = 16) from all farms. There was no correlation found using Spearman’s rank correlation coefficient (−0.16; *p*-value = 0.55)).

## 4. Discussion

This study presents a novel and objective strategy for monitoring the efficacy of RAMPs conducted in herds engaged in a Johne’s disease control programme for reducing the risk of transmission of MAP within herds. The MAP-infected herds in this study were all registered in the IJCP, and engaged in annual RAMPs conducted by a veterinary practitioner. Therefore, it may be concerning that 46% of the herds had detectable MAP in either their calving pen, calf pen, or both locations, particularly if one assumes the bedding material usually present in these areas reduces the sensitivity of environmental sampling for MAP compared to lactating cow housing. While a degree of contamination in the calving area would be expected and accepted as inevitable in infected herds, it is concerning that 21% of herds had detectable levels of MAP in the pre-weaned calf area. The median aWHP across all herds was relatively low at 3.5%, however, the proportion of truly infected animals may be much higher than this, due to the low sensitivity of serology tests for MAP [25]. Another potential factor contributing to the high proportion of positive results could be that the biocontainment measures practiced by the farmers were inadequate, allowing contamination with MAP either through direct shedding from infectious animals (most likely in the calving area) or indirectly through fomites (most likely in pre-weaned calf area), which may include the farmers’ boots. On a typical Irish spring-calving farm the compact calving season may increase the chances of contamination of calf pens with faecal material from adult cows due to the frequent movements of animals and personnel between cow housing (including the calving pen) and the calf rearing areas.

Similar rates of positivity in calving areas and calf pens have been demonstrated in other studies. Pillars et al. (2009) reported finding MAP in the calving area and pre-weaned calf area on 4/6 and 3/6 infected farms that were sampled longitudinally over four years [26]. The herds had annual RAMPs conducted for the duration of the study. The study also found significant associations between increasing within-herd prevalence and the number of positive samples obtained in the calving area and the pre-weaned calf area. Another study of the environmental distribution of MAP in herds enrolled in a Johne’s disease control programme in the US found that 24% of calving pens were positive among 68 farms that had positive environmental samples, however none of the farms had detectable MAP in the pre-weaned calf area [27].

In the present study the relationship between PCR test result and aWHP or RAMP scores at farm-level, location-level or sample-level was not significant. This may be due to insufficient statistical power, and/or the limitations of using RAMP scores or aWHP to assess transmission risk. As mentioned previously, aWHP based on serology likely under-estimates the true proportion of infected animals in the herd that may be contributing to contamination, while RAMP scores are limited by the subjective, point-in-time nature of the assessment [28]. In seasonal calving systems, as the majority of Irish dairy herds are, RAMP assessments are often conducted outside of the busy calving season when the calving pen and calf pens are more likely to be empty. This makes it more difficult for the veterinarian to conduct an accurate assessment of what the transmission risk is, with consequent subjectivity and high inter-observer variability affecting RA scores [29].

The moderate negative correlation (*p* = 0.08) found between the lowest Ct value obtained at a location and the RAMP score for the location suggests that higher RAMP scores may be associated with higher concentrations of MAP in a sample (a lower Ct value). The required significance level (*p* = 0.05) was not achieved, most likely due to low statistical power due to the small sample size and possibly the limitations of the RAMP as discussed above. Factors that may contribute to higher MAP concentrations in an environmental sample from a calving pen, for example, include the number of shedding animals present and the amount of MAP each animal is shedding, as well as the level of hygiene maintained in the pen. The RAMP directly assesses the latter factor, while indirect assumptions about the level of shedding in the pen can be made using aWHP and stocking density. Clumping of MAP bacteria in a sample can also affect the quantification of MAP bacteria during analysis [30]. Bedding material usually present in calving pens and calf pens also likely affects the sensitivity of environmental sampling, however most literature evaluating the sensitivity of environmental sampling for MAP has been focused on main cow housing and manure storage. Pillars et al. (2009) reported that 7% and 14% of calf pen floor and calving pen floor samples, respectively, were culture-positive for MAP, compared to 44% of lactating cow floor samples on seven infected dairy farms.

A limitation of the present study is the small number of farms that were sampled, which likely impacted the significance of the modelling results. A larger study, with longitudinal sampling, would provide further insight into the factors that affect contamination of the pre-weaned calf area and calving area on infected farms.

## 5. Conclusions

This research has demonstrated a potential novel application for environmental sampling as an objective test to complement the annual RAMP, to help monitor the progress of infected farms in reducing the risk of transmission of MAP to calves. However further research conducted on a larger scale is needed to support the results reported in the present study.

## Figures and Tables

**Table 1:** Summary of PCR results, RAMP scores and apparent within-herd prevalence (aWHP) for 28 study farms.

Farm	Pre-Weaned Calf Area PCR Result	Pre-Weaned Calf Area Ct Value *	Pre-Weaned Calf Area RAMP SCORE /80	Calving Area PCR Result	Calving Area Ct Value *	Calving Area RAMP Score /80	Total FarmRAMP Score /227	aWHP (%)
1	Positive	35.76	41	Negative	0	50	131	8
2	Negative	0	44	Negative	0	41	125	21
3	Positive	31.48	32	Positive	31.56	35	104	8
4	Negative	0	29	Inconclusive	38.27	29	119	5
5	Negative	0	23	Negative	0	23	68	1
6	Inconclusive	40.51	17	Positive	31.1	26	74	2
7	Negative	0	20	Negative	0	29	83	3
8	Inconclusive	37.15	14	Positive	33.43	32	83	6
9	Negative	0	11	Negative	0	38	80	1
10	Inconclusive	39.41	26	Positive	33.17	26	104	1
11	Inconclusive	37.65	32	Positive	29.95	47	134	15
12	Negative	0	23	Positive	35.08	35	83	2
13	Positive	32.77	53	Positive	29.52	41	155	4
14	Negative	0	20	Negative	0	23	68	2
15	Negative	0	32	Inconclusive	39.34	32	98	8
16	Positive	34.34	20	Positive	32.68	41	107	2
17	Negative	0	-	Negative	0	-	-	-
18	Negative	0	29	Negative	0	17	71	3
19	Positive	35.66	11	Inconclusive	36.15	38	80	5
20	Negative	0	35	Negative	0	38	125	0
21	Negative	0	20	Negative	0	23	59	4
22	Negative	0	8	Positive	33.78	29	59	6
23	Inconclusive	36.69	23	Negative	0	35	92	10
24	Negative	0	23	Negative	38.09	44	98	3
25	Positive	35.55	32	Inconclusive	41.1	23	98	5
26	Negative	0	35	Inconclusive	40.43	56	155	2
27	Negative	0	23	Negative	0	38	101	-
28	Inconclusive	41.05	11	Positive	35.88	20	47	2

* Ct value reported for each location refers to the lowest Ct value recorded for an individual sample obtained at the location.

**Table 2:** Distribution of pooled PCR results for pre-weaned calf pens and calving pens.

	Positive	Inconclusive	Negative	Total
Calf pens	6	6	16	28
Calving pens	10	5	13	28
Total	16	11	29	56

**Table 3:** Summary of outcomes of logistic regression models with different dependent variables: (1) Farm-level PCR result; (2) Pre-weaned calf area PCR result; (3) Calving area PCR result; (4) Sample-level PCR result (includes both pre-weaned calf area and calving pen samples).

**(1) Farm-Level**				
	Estimate	Odds Ratio	95% CI	*P*-Value
(Intercept)	−0.60395	0.54665	0.024, 11.11	0.692975
log(aWHP + 1)	0.422476	1.525734	0.477, 5.508	0.483722
Overall RAMP	−0.00051	0.999494	0.971, 1.029	0.971782
**(2)** **Pre-weaned calf area only**				
	Estimate	Odds Ratio	95% CI	*P*-value
(Intercept)	−3.42849	0.032436	0.001, 0.584	0.034072
Calf pen RAMP	0.06061	1.062484	0.97, 1.185	0.211207
log(aWHP + 1)	0.348346	1.416723	0.36, 6.733	0.626936
**(3) Calving area only**				
	Estimate	Odds Ratio	95% CI	*P*-value
(Intercept)	−0.34837	0.705836	0.029, 16.388	0.825372
Calving pen RAMP	−0.00639	0.993632	0.907, 1.083	0.883681
log(aWHP + 1)	0.059468	1.061271	0.314, 3.697	0.922076
**(4) Sample level**				
	Estimate	Odds Ratio	95% CI	*P*-value
(Intercept)	−2.07968	0.12497	0.004, 1.512	0.118357
Overall RAMP	0.008885	1.008925	0.984, 1.038	0.450377
log(aWHP + 1)	0.226056	1.253646	0.447, 4.114	0.641828

## Data Availability

The data presented in this study are available in article.

## References

[1] Whittington R.J., Marshall D.J., Nicholls P.J., Marsh I.B., Reddacliff L.A. (2004). Survival and dormancy of Mycobacterium avium subsp. paratuberculosis in the environment. Appl. Environ. Microbiol..

[2] Sweeney R.W. (2011). Pathogenesis of Paratuberculosis. Vet. Clin. N. Am. Food Anim. Pract..

[3] Garcia A.B., Shalloo L. (2015). Invited review: The economic impact and control of paratuberculosis in cattle. J. Dairy Sci..

[4] McAloon C.G., Whyte P., More S.J., Green M.J., O’Grady L., Garcia A., Doherty M.L. (2016). The effect of paratuberculosis on milk yield—A systematic review and meta-analysis. J. Dairy Sci..

[5] Ott S.L., Wells S.J., Wagner B.A. (1999). Herd-level economic losses associated with Johne’s disease on US dairy operations. Prev. Vet. Med..

[6] Waddell L.A., RajiĆ A., StÄRk K.D.C., McEwen S.A. (2015). The zoonotic potential of *Mycobacterium avium* ssp. paratuberculosis: A systematic review and meta-analyses of the evidence. Epidemiol. Infect..

[7] Field N.L., McAloon C.G., Gavey L., Mee J.F. (2022). Mycobacterium avium subspecies paratuberculosis infection in cattle—A review in the context of seasonal pasture-based dairy herds. Ir. Vet. J..

[8] Geraghty T., Graham D.A., Mullowney P., More S.J. (2014). A review of bovine Johne’s disease control activities in 6 endemically infected countries. Prev. Vet. Med..

[9] Groenendaal H., Nielen M., Jalvingh A.W., Horst S.H., Galligan D.T., Hesselink J.W. (2002). A simulation of Johne’s disease control. Prev. Vet. Med..

[10] Kudahl A.B., Østergaard S., Sørensen J.T., Nielsen S.S. (2007). A stochastic model simulating paratuberculosis in a dairy herd. Prev. Vet. Med..

[11] Doré E., Paré J., Côté G., Buczinski S., Labrecque O., Roy J.P., Fecteau G. (2012). Risk factors associated with transmission of Mycobacterium avium subsp. paratuberculosis to calves within dairy herd: A systematic review. J. Vet. Intern. Med..

[12] McAloon C.G., Roche S., Ritter C., Barkema H.W., Whyte P., More S.J., O’Grady L., Green M.J., Doherty M.L. (2019). A review of paratuberculosis in dairy herds—Part 1: Epidemiology. Vet. J..

[13] Khol J.L., Wassertheurer M., Sodoma E., Revilla-Fernandez S., Damoser J., Oserreicher E., Dunser M., Kleb U., Baumgartner W. (2013). Long-term detection of Mycobacterium avium subspecies paratuberculosis in individual and bulk tank milk from a dairy herd with a low prevalence of Johne’s disease. J. Dairy Sci..

[14] Stabel J.R., Bradner L., Robbe-Austerman S., Beitz D.C. (2014). Clinical disease and stage of lactation influence shedding of Mycobacterium avium subspecies paratuberculosis into milk and colostrum of naturally infected dairy cows. J. Dairy Sci..

[15] Whittington R., Donat K., Weber M.F., Kelton D., Nielsen S.S., Eisenberg S., Arrigoni N., Juste R., Saez J.L., Dhand N. (2019). Control of paratuberculosis: Who, why and how. A review of 48 countries. BMC Vet. Res..

[16] Ridge S., Baker I., Hannah M. (2005). Effect of compliance with recommended calf-rearing practices on control of bovine Johne’s disease. Aust. Vet. J..

[17] Sorge U., Kelton D., Lissemore K., Godkin A., Hendrick S., Wells S. (2010). Attitudes of Canadian dairy farmers toward a voluntary Johne’s disease control program. J. Dairy Sci..

[18] Gavey L., Citer L., More S.J., Graham D. (2021). The Irish Johne’s Control Programme. Front. Vet. Sci..

[19] Raizman E.A., Wells S.J., Godden S.M., Fetrow J., Friendshuh K., Michael Oakes J. (2006). Characterization of Minnesota dairy herds participating in a Johne’s disease control program and evaluation of the program risk assessment tool. Prev. Vet. Med..

[20] Donat K., Hahn N., Eisenberg T., Schlez K., Köhler H., Wolter W., Rohde M., Pützschel R., Rösler U., Failing K. (2016). Within-herd prevalence thresholds for the detection of Mycobacterium avium subspecies paratuberculosis-positive dairy herds using boot swabs and liquid manure samples. Epidemiol. Infect..

[21] Eisenberg T., Wolter W., Lenz M., Schlez K., Zschock M. (2013). Boot swabs to collect environmental samples from common locations in dairy herds for Mycobacterium avium ssp paratuberculosis (MAP) detection. J. Dairy Res..

[22] Caldwell D.J., Hargis B.M., Corrier D.E., DeLoach J.R. (1998). Frequency of Isolation of Salmonella from Protective Foot Covers Worn in Broiler Houses as Compared to Drag-Swab Sampling. Avian Dis..

[23] Talorico A.A., Bailey M.A., Munoz L.R., Chasteen K.S., Pal A., Krehling J.T., Bourassa D.V., Buhr R.J., Macklin K.S. (2021). The use of roller swabs for Salmonella detection in poultry litter. J. Appl. Poult. Res..

[24] Wolf R., Barkema H.W., De Buck J., Slomp M., Flaig J., Haupstein D., Pickel C., Orsel K. (2014). High herd-level prevalence of Mycobacterium avium subspecies paratuberculosis in Western Canadian dairy farms, based on environmental sampling. J. Dairy Sci..

[25] Nielsen S.S., Toft N. (2008). Ante mortem diagnosis of paratuberculosis: A review of accuracies of ELISA, interferon-γ assay and faecal culture techniques. Vet. Microbiol..

[26] Pillars R.B., Grooms D.L., Kaneene J.B. (2009). Longitudinal study of the distribution of Mycobacterium avium subsp paratuberculosis in the environment of dairy herds in the Michigan Johne’s disease control demonstration herd project. Can. Vet. J. Rev. Vet. Can..

[27] Raizman E.A., Wells S.J., Godden S.M., Bey R.F., Oakes M.J., Bentley D.C., Olsen K.E. (2004). The distribution of Mycobacterium avium ssp paratuberculosis in the environment surrounding Minnesota dairy farms. J. Dairy Sci..

[28] McAloon C.G., Whyte P., More S.J., O’Grady L., Doherty M.L. (2015). Development of a HACCP-based approach to control paratuberculosis in infected Irish dairy herds. Prev. Vet. Med..

[29] Pieper L., DeVries T.J., Sorge U.S., Godkin A., Hand K.J., Perkins N.R., Imada J., Kelton D.F. (2015). Variability in Risk Assessment and Management Plan (RAMP) scores completed as part of the Ontario Johne’s Education and Management Assistance Program (2010–2013). J. Dairy Sci..

[30] Elguezabal N., Bastida F., Sevilla I.A., González N., Molina E., Garrido J.M., Juste R.A. (2011). Estimation of Mycobacterium avium subsp. paratuberculosis growth parameters: Strain characterization and comparison of methods. Appl. Env. Microbiol..

